# Fish diversity assessment through conventional morphological identification and recent advances in Saudi Arabia: A review

**DOI:** 10.14202/vetworld.2024.2267-2285

**Published:** 2024-10-07

**Authors:** Muhammad Browijoyo Santanumurti, Muhammad Ar Rozzaaq Nugraha, Novi Rosmala Dewi, Muhammad Awaluddin, Pei Wen Tang, Helen Indah Pardede, Lafi Al Solami, Laksmi Sulmartiwi, Mohamed Ahmed Abu El-Regal

**Affiliations:** 1 Department of Aquaculture, Faculty of Fisheries and Marine, Universitas Airlangga, Surabaya, 60115, Indonesia; 2 Department of Marine Biology, Faculty of Marine Sciences, King Abdulaziz University, Jeddah, 21589, Kingdom of Saudi Arabia; 3 Department of Aquaculture, National Taiwan Ocean University, Keelung, 20224, Taiwan, ROC; 4 Department of Bioscience and Biotechnology, National Taiwan Ocean University, Keelung, 20224, Taiwan, ROC; 5 Department of Marine, Faculty of Fisheries and Marine, Universitas Airlangga, Surabaya, 60115, Indonesia; 6 Department of Marine Science, Faculty of Science, Port Said University, Port Said, 42526, Egypt

**Keywords:** fish diversity, identification process, Saudi Arabia

## Abstract

Fish identification in the Red Sea, particularly in Saudi Arabia, has a long history. Because of the vast fish diversity in Saudi Arabia, proper species identification is required. Indeed, identifying fish species is critical for biodiversity conservation, food and drug safety, and sustainable fishery management. Numerous approaches have been used to identify fish species, including conventional morphological identification, next-generation sequencing (NGS), nanopore sequencing, DNA barcoding, and environmental DNA analysis. In this review, we collected as much scientific information as possible on species identification in Saudi Arabia. Our findings suggest that the identification process has advanced and spread rapidly and broadly, as evidenced by the discovery of new fish species in Saudi Arabia. The advantages and disadvantages of each method were discussed as part of a comprehensive comparison. This study aimed to provide further scientific knowledge to promote the growth of fish diversity worldwide.

## Introduction

**Figure-1:**
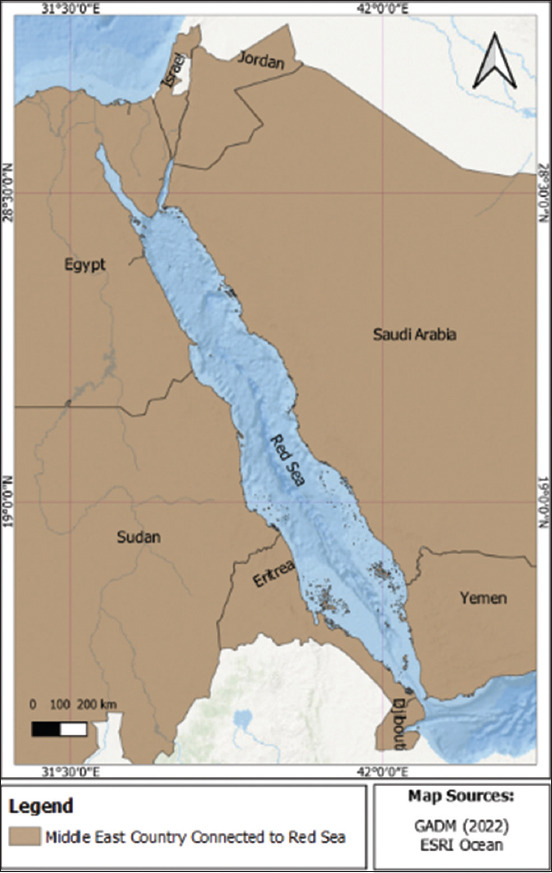
Location of the Red Sea surrounded by eight countries.

The Red Sea has substantial potential for marine fishery biodiversity (Figure-1). The Red Sea rises from Suez to the Bab-el-Mandeb Strait, with a length of more than 2000 km and a width of 200–300 km [1]. Uniquely, the Red Sea borders eight countries and is famous for its high fish biodiversity owing to its coral reef area of up to 16,000 km^2^. More than 1100 fish species have been identified in the Red Sea, representing only 12.9% of endemic species [2, 3]. Among the Red Sea countries, Saudi Arabia borders and covers most of the eastern Red Sea Basin. Saudi Arabia has a total coastline of 7572 km, including the Red Sea (west) and the Arabian Gulf (east) [4]. The ecosystem of the coast of Saudi Arabia is characterized by vibrant natural environments populated by many forms of marine life. Over one-third of all known fish species spend part or all of their lives in coral reef habitats [5]. The country has approximately 390 scleractinian coral and 1078 fish species [1, 3]. The total amount of fish caught in the Red Sea in the Kingdom of Saudi Arabia was 24,016 metric tons in 2018 [5]. These results demonstrate that the potential for marine fish diversity in the waters of Saudi Arabia is high.

The proper identification of fish species is necessary because of the enormous diversity of fish in Saudi Arabia. Although research on fish identification continues, one prior study proposed that 91% of oceanic species, including fish, have not yet been discovered [6]. This identification is essential for maintaining biodiversity (evolutionary biology, interaction, and presence of endangered species), food and drug safety (ingredients and sources), and sustainable fishery management (estimating fish density and stock status) [7–9]. Conventional fish species identification has been performed in Saudi Arabia since 1761 [10]. At present, several assessment techniques are used to identify fish species, of which next-generation sequencing (NGS) and DNA barcoding represent important advances in this field. NGS uses massively parallel sequencing methodologies to generate millions of short-read sequences in a significantly shorter time and with higher throughput than Sanger sequencing (first-generation sequencing) [11]. DNA barcoding is a technique commonly used to identify organisms based on tissue DNA sequences from tissues [12]. This method is superior and more accurate than the conventional morphological identification methods (meristic and morphometric), which are limited by their heavy dependence on taxonomic information (books and other publications) and the taxonomist’s experience (user or person who observes the species) [13]. The absence of these two factors affects the accuracy of the identification process, which will undoubtedly affect fishery management and biodiversity. In addition, fish include cryptic and alien species that are not easily identified visually [13, 14].

This review discusses fish assessment techniques, their advantages, disadvantages, and applications in Saudi Arabia. This review will also benefit academics working in this sector regarding experimental design, understanding the results obtained, and providing essential tools for future research, particularly in Saudi Arabia.

## History of Fish Diversity in Saudi Arabia

The rich diversity of fish in the Red Sea has attracted the attention of researchers worldwide. Peter Simon Forsskål conducted the first expedition to the Red Sea in 1761 [15]; on the same expedition, a researcher from Finland brought six Danish scientists with him on the expedition [16]. Unfortunately, Forsskål and five other scientists died of malaria upon their arrival in Yemen [17]. Niehbuhr, the only survivor of the first expedition to the Red Sea, published Forsskål’s findings in 1775, containing 122 reported species from the Red Sea [18]. The French army also reported the results of fish assessment in the Red Sea during its second expedition, from 1798 to 1801 [19]. The results of the expedition were published under the title “Histoire naturelle des poissons de la Mer Rouge et de la Méditerranée (Natural History of the Fishes of the Red Sea and the Mediterranean)” by Isidore Geoffroy Saint-Hilaire (1817 and 1829) [19].

Research on the Red Sea continued into the 1800s by German ichthyologist Wilhelm Friedrich Hemprich as well as by botanist Christian Gottfried Ehrenberg from 1820 to 1826 [10]. Hemprich and Ehrenberg collected many animal and plant specimens and more than 500 fish species [16]. However, their joint expedition was cut short in 1825 when Hemprich died of malaria, whereupon Ehrenberg returned to Berlin with his collected material. In addition to Hemprich and Ehrenberg, Eduard Rüppell explored the Red Sea from 1811 to 1836 [10]. In his first publication, Rüppell described 161 fish species (Rüppell, 1828), whereas in his second book (Rüppell, 1835–1838), he described 164 fish species, of which approximately 100 were new to science [16]. Carl Benjamin Klunzinger, an ichthyologist from Germany, played an important role in fish identification in the Red Sea from 1864 to 1884 [10]. Klunzinger first attempted to compile a list of all the known fish species in the Red Sea. Klunzinger’s lists were published in a reprint and revised with another 101 species in 1877 [20].

Starting in the 1990s, the research on Red Sea species became significantly more comprehensive. In 1971, Botros attempted to compose a complete checklist of all Red Sea fish, compiling a list of approximately 750 species [21]. Unfortunately, his limited background in ichthyology prevented him from including taxonomic changes accepted since the publication of Klunziger’s synopsis. However, his publications accurately present the history of scientific expeditions to the Red Sea. Bogorodsky and Randall [16] stated that Dor made the compilation of an accurate checklist of the Fishes of the Red Sea (CLOFRES), with close to 1000 reported species. A decade later, Bogorodsky and Randall [16] stated that Goren and Dor published CFLORES II, the updated list of Red Sea fishes original checklist, with nearly 250 species added. The concepts used in CLOFRES and CLOFRES II include all records, quotations, and distribution maps, without distinguishing between substantive and doubtful records. Subsequently, many researchers attempted to compile and discover new species in the Red Sea. One report stated that 1120 coastal fish had been found in the Red Sea, accounting for 14.6% of all endemic species [16]. FishBase, a biodiversity information system on finfish, showed that 895 species of fish had been reported in Saudi Arabia. The fish were found in the Red Sea and Arabian Gulf, located on the eastern side of the Kingdom; however, it should be noted that the Red Sea covers three times the area of the Arabian Gulf. Fish identification using morphological and molecular methods has recently been performed in Saudi Arabia. Morphological identification has been informally performed in Saudi Arabia since the 1980s [22–24]. Before the 1950s, a report showed that the people of Saudi Arabia who worked as fishermen were familiar with the fish species they caught [25].

Trivedi *et al*. [26] published the first report on DNA barcoding experiments in Saudi Arabia. Although the Red Sea is rich in fish biodiversity, no such studies have been previously reported. This study reported the cytochrome oxidase subunit I (*COI*) genes of six fish species (*Epinephelus chlorostigma*, *Siganus rivulatus*, *Carangoides bajad*, *Scomberomorus commerson*, *Lutjanus ehrenbergii*, and *Pristipomoides filamentosus*) from the coastal waters of Tabuk, Saudi Arabia. Two novel sequences were identified from the six fish samples collected. Rabaoui *et al*. [24] described the high diversity of fish in the Gulf of Saudi Arabia, reporting that this area contained 200–550 fish species. However, a knowledge gap in DNA barcoding remains, as only a few studies have been conducted on molecular identification. The species found in the Saudi Arabian Gulf were identical to those reported for the same species in the Indo-West Pacific Ocean. In addition to molecular identification using the *COI* gene, a previous report by Shaikh-Omar *et al*. [27] identified *Epinephelus* species (*Epinephelus areolatus*, *Epinephelus malabaricus*, *Epinephelus summana*, *Epinephelus radiates*, and *Epinephelus chlorostigma*) using the *Otx1B* gene. However, the use of *Otx1B* is not as widespread as that of *COI*, as the former can distinguish between close and cryptic species [12]. The *COI* gene is a powerful tool for identifying fish species, and it has been applied in various studies [28, 29]. This gene lacks introns and has a high copy number, while its maternal inheritance makes it a good candidate for DNA barcoding [30].

## Pivotal Background of Conventional Morphological Identification

An increasing number of species are transported worldwide and traded in continents far from their origins, increasing the need for global fish identification tools to provide reliable information to consumers, customs officials, and fishery inspectors. However, there are more than 32,500 species of finned fish worldwide, and the quantity of information needed to separate them is extremely difficult to handle; as such, fish identification is generally performed on a local or regional scale [31, 32].

**Figure-2:**
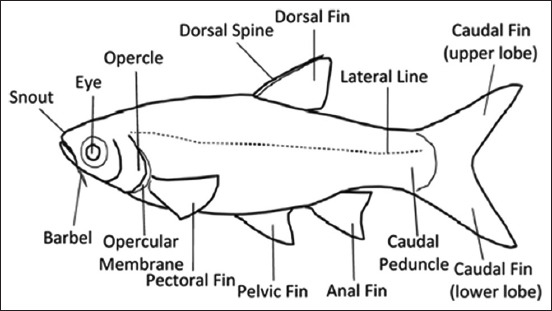
Primary morphological traits of the *Cyprinidae* species [33].

**Figure-3:**
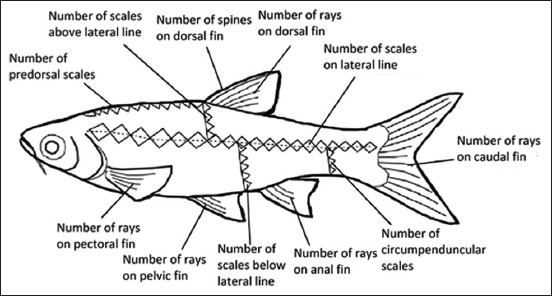
Typical morphometric data for fish identification [33].

Conventionally, fish identification has been based on morphological characteristics. Ng *et al*. [33] presented images for the morphological identification of fish (Figures-2 and 3). However, owing to their high diversity and morphological flexibility, fish and their different developmental stages are often difficult to identify using morphological characteristics alone. Recently, public awareness of the need to conserve biodiversity has increased. To this end, policymakers, funding agencies, and scientists have prioritized the advancement of policies and knowledge, an interest sparked by the realization that taxonomic resources worldwide are rapidly depleting and harming the well-being and survival of humans.

According to the International Code of Zoological Nomenclature, a stable naming and indexing system is essential for the global communication of such organisms and systems. Taxonomy provides the methods and guidelines for the identification of organisms. Basic taxonomic tools used in fisheries include FishBase [34], the book Fish Species of the Northeast Atlantic and Mediterranean, a series of catalogs and checklists, and a regional survey provided by the Food and Agriculture Organization. Although the study, mapping, taxonomic characterization, and naming of the world’s marine animals and freshwater fish are fundamental to healthy fisheries, the importance of taxonomy remains unclear in the fishing sector, particularly in northern regions where “everything is known.”

This review covers the most commonly used methods for identifying aquatic species, including traditional, reliable, and long-tested tools such as qualified taxonomists, reference collections, and field guides based on course content. Dichotomy and recently developed tools are commonly used in experimental identification systems (image recognition systems [IRS]), interactive electronic keys, computer-based morphometric identification (IPez), and genetic methods. Local expertise, scales, otoliths, and (popular) hydroacoustics have also been considered. However, some methods have not been evaluated in detail as they are too general for identifying fish through image browsing (using the web) or because they have limited applications, such as using vertebrates.

## ID-tool: Local (Folk)

Expert folk taxonomy is a classification system created by non-scientists to organize, name, and understand the natural world. Popular classifications often deviate from the phylogeny established by scientific taxonomic studies in some respects; however, they also tend to conform to scientific classifications in other respects. Common taxonomic groups collect many biological species under a single name or place species from different biological orders within the same group. Sometimes, popular and scientific taxonomies show a one-to-one correspondence, while in some other instances, popular taxonomies help distinguish where scientific taxonomies do not. The differences between types in common taxonomies can be determined by many attributes, some of which are not immediately obvious to outsiders.

Understanding morphology and behavior is essential, as well as recognizing the cultural significance and practical utility of the species that comprise each group. Common classifications thus not only reflect how people observe components of the environment but also relate to their perceptions and understanding of the natural system as a whole [35]. People can make biological inferences about an organism based on other organisms they perceive to be similar [36, 37]. The consequent discrepancies between scientific classification systems and popular perceptions of biodiversity can lead to mismatch between the scientific and stakeholder perspectives.

Understanding how stakeholders perceive biodiversity as significant in ecosystems inaccessible or unobservable to most people and where successful conservation depends on the active voluntary participation of relevant parties is important. For example, compliance with species-selective harvest regulations and the accuracy of harvest data collected by natural resource agencies both depend on the ability of fishers and hunters to identify the managed species [38]. In addition, species naming, classification, and identification generate public support for conservation [39, 40]. As such, gaining widespread support for the recovery of a species morphologically similar to other species or which is unfamiliar to stakeholders may be difficult.

## Local Reference Collection

Reference collections comprise preserved specimens of whole fish, otoliths, disarticulated bones, scales, pharyngeal bones, and similar body parts used to identify species. Local reference collections are primarily found in research institutions (and fishery agencies) and are generally dedicated either to a restricted geographical area or a particular research purpose. Local reference collections may be sufficient for identification work in restricted areas and to reduce the need for expert consultations, keys, field guides, and other methods. These tools are beneficial for smaller institutions in field-like situations and can further be used to train new staff continuously.

To understand its cultural context and interrelationships, a local reference collection is best conceptualized as a body or system of knowledge rather than a mere assemblage of facts. First, it involves understanding how a local reference collection, including related skills, is communicated and transmitted *in situ* and *in vivo* as part of the exigencies of maritime life [41–52]. Local reference knowledge on habitats, species, and the relationships between these two variables is collected at the level of the individual fisher while also exploring fishers’ mental schemata of habitats and the habitat of each species, coupling a specific example of the mutton snapper as a prototype [53]. The relationship between a fisherman’s ecological knowledge and fishing success has been investigated, with research showing that human factors, such as knowledge and skill, may play as much of a role in fishing success as material or technological factors [54].

The observation of specimen anatomy to distinguish fish species based on physical characteristics is the most practical, rapid, and inexpensive method. In addition to expert local fishermen and fishmongers, individuals living near rivers or wetlands often learn to recognize fishes early thanks to the knowledge and memories acquired through long-term observations or oral traditions passed down by the elderly. Many researchers have incorporated traditional knowledge into contemporary ichthyology [55], which is referred to as “traditional ecological knowledge” [56]. It is necessary for people interested in taxonomy to embrace them.

The identification of the placement of fins and the number and type of ray or spine components is essential in the morphometric and meristic identification of fish. Morphological and meristic database compilations can be rapidly peer-reviewed and shared online by amateurs and specialists worldwide. This represents an opportunity for scientists to connect with society and gain support from the public and politicians for fish conservation.

## IRS

In this method, the user provides a photograph (image) of the fish as input and the software (IRS) identifies the fish to a taxonomic level. The identification process is based on the automatic characterization of visual image properties, such as color, texture, and shape, using computer vision techniques, that is, image retrieval and classification approaches that exploit feature vectors and similarity functions. Image-processing methods are commonly used to encode visual properties into feature vectors, while similarity functions are used to compute the similarity between two images by considering their feature vectors.

Studies on fish image recognition are particularly important in marine biology and aquaculture. Fish generally have a skull and spine and breathe through gills attached to the skin. They most commonly have a slender body shape suitable for swimming and fins to allow them to move faster through water. Fish can be categorized into two types: saltwater and freshwater. Looking at mechanical and artificial classifications, artificial classification causes eye fatigue and low efficiency and represents an enormous workload in the face of many fish classifications [57]. In contrast, mechanical classification of fish improves work efficiency [31] but causes enormous damage to fish. Based on the shortcomings mentioned above, this paper presents a new form of fish recognition based on image processing and statistical technology research, which predominantly relies on image processing techniques using MATLAB Software (https://www.mathworks.com/products/matlab.html) for fish image processing and analysis [58]. Further, a principal component analysis method to characterize the characteristics of dimension reduction has been proposed [32, 59]. The Fisher discriminant method with a Markov distance mathematical model has also been established to classify and verify four types of fish. The results showed that this technology has significant economic and application value. Image acquisition was performed in this technique, with images of four fish (chub, crucian, and bream fish). Color images in JPG format were obtained using a digital camera and were transferred to the computer, whereupon, to obtain the basic information of the image, MATLAB Software was used to analyze and gather information. Image preprocessing was performed to improve the quality of images, extract the fish features, and improve the accuracy of image information. The preprocessing stage comprised image gray-level processing, linearization, enhancement, and contour extraction. Extraction of color, texture, and shape features was also performed as preparation.

## Field Guides Based on Dichotomous Keys

Diagnostic taxonomic keys are a common, traditional means of identifying organisms and are essential to most field guidelines. A taxonomic key is defined as an ordered sequence of choices provided by organisms’ diagnostic (morphological) characteristics that allow the reliable identification of an organism or class of organisms. Diagnostic characteristics are defined in a key, which can be illustrated for clarity. A key’s formal or taxonomic scope is generally restricted to printed materials or presentations in digital format [34].

## Pivotal Background of Illumina NGS

Illumina NGS is a powerful parallel sequencing technology that has been available since the beginning of the 21^st^ century [60]. NGS has been extensively applied in aquaculture and fisheries to investigate disease [61], breeding, and genetics [62, 63], with lower costs than Sanger sequencing. In general, NGS involves several significant steps: (i) DNA or RNA extraction, (ii) library preparation, (iii) biotechnological sequencing, and (iv) data analysis and interpretation [64]. It offers ultrahigh throughput and rapid screening or reading of millions of DNA or RNA sequences, generating vast sequencing data that provide comprehensive information for “omics” studies, such as genomics, transcriptomics, proteomics, and metabolomics [65, 66]. Each application provides information on the genome structure, gene expression profiles, and gene function of the different organisms [67], allowing for an in-depth understanding of the molecular mechanisms governing various biological and physiological processes [68].

## Illumina NGS for Genomic Analysis

The basic definition of “genomics” in biology is a study focused on gene structure, function, evolution, or mapping of genomes [69, 70]. Whole-genome sequencing (WGS) using Illumina NGS technology is a popular genomic method involving the analysis of DNA sequences to identify gene functions and their involvement in various fields [71–73]. To enhance aquaculture production efficiency and sustainability, WGS has been used to improve the traits of fish and other aquatic animals, such as promoting rapid growth, product quality, disease resistance, and tolerance to diverse environmental stressors, to meet the needs of human consumption in the near future [74].

One case study of the application of WGS in tilapia (Oreochromis spilurus) identified 51,642 protein-coding peptides that play a role in major cellular processes and several diseases in fish, making tilapia a prominent species in aquaculture in Saudi Arabia [75]. This genomic investigation of this tilapia subspecies suggested that these fish possess a high saltwater tolerance gene, allowing them to survive easily in the Red Sea of Saudi Arabia, which has a salinity of 42 ppt. In addition, aquaculture diseases pose a significant challenge and pressing concern for the sustainable development of aquaculture. White spot syndrome virus has been detected in >95% of fatalities in penaeid shrimp aquaculture in Saudi Arabia [76, 77]. Applying WGS to various diseases may provide novel insights into enhancing disease resistance in various aquaculture species in Saudi Arabia.

## Illumina NGS Transcriptomic Analysis

Transcriptomic analysis, also known as RNA-sequencing (RNA-seq) in aquaculture, covers several areas of interest, including immunology, response to stressors, sexual dimorphism, and development [78]. It refers to a complete set of transcripts, including protein-coding messenger RNA, non-coding RNA such as ribosomal RNA, and transfer RNA [79, 80]. In recent years, transcriptomic studies have provided a better understanding of biological systems in different fish species, primarily focusing on environmental effects such as alkalinity, temperature, salinity, and ammonia, as well as immunological responses to diseases, such as the physiological response to starvation and rearing density [81, 82]. These studies have been performed to examine the gene expression patterns of control and experimental treatment (co-occurrence of hypoxia and high pCO_2_ or exposure to pollutants) [83, 84], investigating which genes are upregulated or downregulated to develop a better understanding of the metabolism of certain nutrients, diseases, stress pathways, as well as the development of specific organs [85]. In general, the three significant steps in transcriptome analysis are complementary DNA library construction, sequencing on a specific NGS platform, and bioinformatics analysis. In addition, de novo transcriptome assembly can be performed using Trinity Software (https://www.ncbi.nlm.nih.gov/pmc/articles/PMC3571712/) when no reference genome or transcriptome is available [86, 87].

One typical case study is that of the Arabian pupfish *Aphanius dispar*, a euryhaline species belonging to the family Cyprinodontidae that is widely spread around the Red Sea along Saudi Arabia’s western coast. In this study, de novo transcriptome assembly demonstrated the potential of Arabian pupfish from near-freshwater habitats to survive and acclimate to higher salinity habitats by comparing the gill gene expression profiles between desert pond fish and Red Sea coastal lagoon fish. The results showed that during short-term acclimation to higher water salinity, cellular stress response processes were triggered to prevent permanent damage to the fish following acute hyperosmotic exposure. However, long-term acclimation revealed that the pathway involved in gill epithelium modification lasted, allowing adaption to the increased salinity [88]. These results illustrated that transcriptome analysis represents an extensive toolkit for molecular processes critical for adaptation to high-stress environments in organisms.

## Nanopore Sequencing

The concept of nanopore sequencing was first proposed in the 1980s, subsequently being developed and refined over the intervening three decades. Nanopores use pores implanted in a membrane that divides into two compartments to sense DNA or RNA bases directly, rather than the widely used sequencing-by-synthesis approach. DNA flows through the pores, while an ion current is generated when an electric potential is applied across the membrane. DNA sequences can be deduced using the unique current signals produced when nucleotides in the pores alter ion flow [89]. Nanopore sequencing involves the application of an electric current to a hole (nanopore) with a diameter of 1 nm through which the DNA sequence can pass. The electrical current flowing through the pores is altered for every nucleotide, and the signal is instantaneously detected [90]. Nanopore sequencing is a promising next-generation DNA and RNA-seq technique, owing to its high speed, single-base sensitivity, and extended read lengths [91]. Measurement of the variations in the electrical signals produced by DNA or RNA molecules moving through nanoscale pores forms the foundation for nanopore sequencing. A conserved gene region with sufficient variation in DNA sequences that enable differentiation between closely related taxa is the target of the molecular technique known as DNA metabarcoding. This technique is typically used in extensive species identification when the source material comprises numerous species. This method is increasingly used in dietary research, where the primary samples are fecal matter, regurgitated food, or gut material [92–96]. Nanopore sequencing can be used to differentiate between individual nucleotides by monitoring changes in electrical conductivity as DNA molecules pass through a pore.

Oxford Nanopore Technologies (ONTs) is the developer and marketer of the third-generation nanopore sequencing technology, which uses a tiny portable sequencing equipment known as MinION (Oxford Nanopore Technologies, Oxford, UK) [97]. The MinION DNA sequencer from ONT, which is smaller than a smartphone and can provide data in minutes, is a promising example of a rapid detection technology. An electrical field allows a low-cost sequencer to sequence individual DNA molecules as they pass through the biological nanopores. This device has many intriguing features, including real-time analysis, an inexpensive initial investment, and long-read sequencing (the maximum read length now reaches 880 kb, with a mean read length that frequently surpasses 10 kb) [97]. GridION/MinION devices from Nanopore Technologies can sequence a single molecule with a longer read length. The amplification stage can be skipped when nanopore sequencing technology is used, as the DNA template can be directly sequenced [98]. However, compared with common NGS equipment, such as the Illumina MiSeq, MinION sequencing exhibits a more significant error rate. Numerous applications, including biodiversity DNA barcoding, have been made possible by nanopore sequencing, allowing the rapid assessment of complete individual mitogenomes, even in species with limited public genetic information. This substantially eliminates the need for species-specific assays or prior knowledge of the species. In addition to producing massive amounts of new data that can be used for screening and other purposes, this technology can further quickly generate enormous sequencing data from extracted DNA, facilitating species identification [99]. Real-time signal detection can be achieved by varying the electrical current flowing through the pores of each nucleotide. Unlike other third-generation sequencing methods, this methodology does not require chemical sample tagging or polymerase chain reaction (PCR) amplification. Accurate sequencing of low-complexity sections is sometimes difficult with nanopore sequencers, as small changes in the electrical signal of the pore occur when the base remains unchanged. A MinION Analysis and Reference Consortium study found that 2D pass readings had a total inaccuracy of 10.5%, with approximately 3% of the errors originating from mismatches and insertions, with more arising from deletions. Accurate length determination is difficult due to variations in the DNA translocation speed. The proposed technology makes it challenging to distinguish between sample and reference genome sequences. Nanopore sequencing is likely to render all other sequencing devices obsolete, as demonstrated by MinION, a portable, pocket-sized device with extended read times allowing real-time base detection (without fluorescent tags) with low sample collection and sequencing costs [90].

The use of nanopores for electrical DNA detection offers significant advantages over fluorescence microscopy and cyclic arrays. Typically, single-molecule sequencing uses a polymerase to enzymatically incorporate a fluorescently-tagged mononucleotide, using radiation suppression techniques to identify a single molecule. Nanopore sequencing is an entirely new method that relies on an electrical signal generated when DNA is translocated through a pore in a membrane rather than fluorescent tagging or any other chemical treatment. A robust nanoporous structure of an appropriate size must be used to sequence DNA using nanopores. The primary equilibrium form of DNA, B-form double-stranded DNA (dsDNA), is a stiff, highly charged polymer with a solvated, helical structure of approximately 2.6–2.9 nm in diameter, which performs neutron scattering depending on the sequence and the number of firmly bound water molecules in the primary hydration shell. Single-stranded DNA (ssDNA) is approximately half the size of this material, and its flexibility thus allows it to fit through the 1-nm pore. The orientation of the polymer segment endures over a distance known as the persistence length, owing to the limited elasticity of DNA. Compared with dsDNA, which has a persistence length of approximately 50 nm, ssDNA has a length of 0.75–4 nm, depending on the quantity of salt. Using α-hemolysin prototypes, the potential for low-cost, high-throughput nanopore sequencing is being investigated [100].

## DNA Barcoding

**Figure-4:**
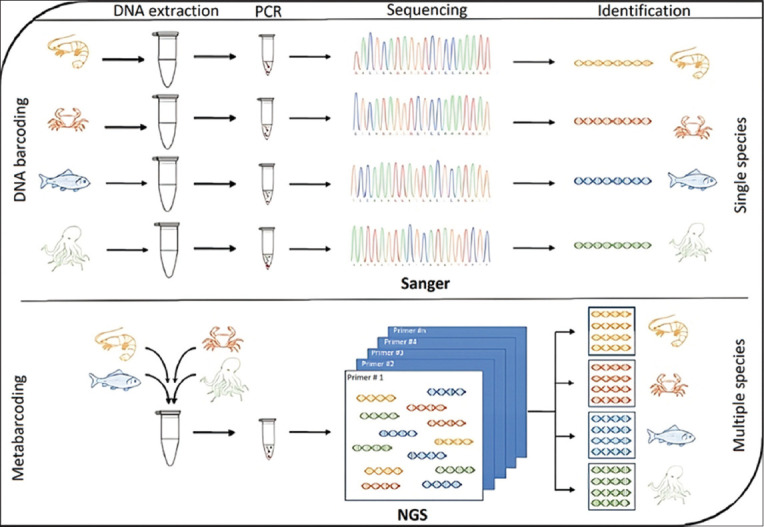
A schematic representation of the essential phases of DNA barcoding and metabarcoding for species identification [103].

DNA barcoding has been used in various biotechnological fields. This technique represents an efficient molecular diagnostic tool for identification (taxonomy), community ecology, conservation biology, and evolution of certain functional traits in organisms [101, 102]. Fernandes *et al*. [103] previously presented a scheme for the essential phases of DNA barcoding for species identification (Figure-4). In particular, fish DNA-barcoding projects have provided comprehensive taxonomic coverage of marine and freshwater environments [104]. In general, DNA barcoding is performed over two significant steps: (i) creating a DNA barcode library of identified species and (ii) matching the DNA barcode sequence of an unidentified sample to the DNA barcode library [105].

The mitochondrial cytochrome c oxidase subunit 1 gene is a short, standardized regional fragment (~648 bp) commonly used as a global marker for species recognition [106]. The *COI* gene is sufficiently short to be sequenced quickly and inexpensively [107]. To date, *COI* has successfully been identified in many types of fish, including tuna, flatfish, anchovy, catfish, and other economic aquatic species [108, 109]. In addition, it has been used by the United States Food and Drug Administration to identify seafood products and investigate seafood-related illnesses relevant to consumers [110]. Following the DNA barcoding protocol for fish, unknown fish species can be collected for DNA extraction and the *COI* gene can be amplified using PCR. The results are then aligned against a reference database for identification [111]. The Fish Barcode of Life campaign (http://www.fishbol.org) is a database established for all fish species, derived from voucher specimens with authoritative taxonomic identification. It is a powerful tool that supports taxonomic classification to accelerate the fish identification process [112–114].

One DNA barcoding case study was performed to investigate species richness in the western and southern areas of the Saudi Arabian Gulf, which hosts 200–500 reported fish species [115]. The results showed that fish species from the Gulf are closely related to species from surrounding waters, including the Red Sea and western Indian Ocean. In this study, the *COI* gene was used as a molecular marker to perform DNA barcoding for Gulf fish, revealing that the technique could be used to manage and preserve fish diversity in the Gulf [24]. The *COI* is a typical barcode found in pufferfish and other fishing-related products. Because cells have more mitochondrial DNA, they have a higher degree of recovery and are heat resistant [116]. Another DNA barcoding study on red sea fish from Saudi Arabia detected and identified two new fish species (E. chlorostigma and S. rivulatus) available in the fish market [26]. These studies demonstrated that DNA barcoding is a practical, reliable, and accurate technique to identify fish and understand their regional and global biodiversity.

## Environmental DNA (eDNA)

**Figure-5:**
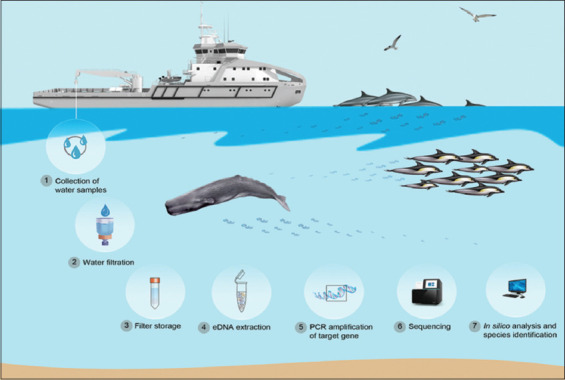
Example of environmental DNA application in marine mammals, including species, biodiversity, and genetic characterization [123].

Originally developed, tested, and applied in the mid-1980s for the detection of bacteria in marine sediments [117], aquatic eDNA applications only began to receive attention in the early 20^th^ century following the performance of studies on fecal pollution in freshwater systems targeting both prokaryotic [118] and eukaryotic communities [119]. In recent years, driven by the emergence of high-throughput sequencing in conceptual, analytical, and technological developments, aquatic eDNA methods have begun to detect invasive species in freshwater [120, 121] and monitor marine mammals [122]. Suarez-Bregua *et al*. [123] described a schematic mechanism for eDNA application in marine mammals (Figure-5) [123], serving as a basis for the identification of other species using the same method. Shortly after these groundbreaking studies, the first comprehensive literature reviews were published in 2012 [124, 125], consolidating the term “eDNA” [126] and introducing further methodological developments to assess fish biomass and improve eDNA detection probability [127]. Over the past decade, eDNA analysis techniques have been developed, tested, and applied to almost all types of aquatic and terrestrial ecosystems [128], including subterranean environments [129], Antarctic geothermal sites [130], coral reefs [131], and deep oceans [132]. These eDNA developments have recently been described as a “quiet revolution transforming conservation,” fostering enormous benefits for biomonitoring and all its derived disciplines in the past decade. eDNA biomonitoring is one of the most effective baseline tools for assessing the environmental effects of numerous anthropogenic and non-anthropogenic stressors.

eDNA is the extra organismal genetic material suspended in environmental samples such as water and sediment. eDNA is shed from macro-organisms through feces, body mucus, blood, and sloughed tissue or scales and has arisen as an alternative data source for biodiversity monitoring [133–136]. When filtering a certain amount of water, eDNA can be captured and concentrated on the filter membrane, from which it is subsequently extracted and subjected to various molecular biology experiments to detect and identify organisms [137–139]. In particular, eDNA metabarcoding enables the simultaneous detection of multiple species using high-throughput NGS platforms [122, 124, 140].

DNA barcoding databases have been established for fish in many regions of the world. However, DNA barcoding in Saudi Arabian Gulf fish is lacking despite the high diversity of ichthyological assemblages in this area. Indeed, although Saudi Arabia is one of the largest and most productive Gulf countries in terms of marine fish production, few studies have been conducted on fish species in Saudi waters, and no inventories of Saudi marine species, including fish, have yet been published. While 200–550 fish species have been reported in the Gulf [115, 141], species richness shows significant regional variation, with a knowledge gap for species in the western and southern areas [142, 143]. One study was therefore conducted to build a database of DNA barcodes for the Saudi Arabian Gulf fish. This study aimed to provide DNA barcodes for 117 marine fish belonging to 54 families and 13 orders collected from Saudi Arabian waters to obtain helpful information for managing and preserving fish diversity in the Gulf.

eDNA-based evaluation of intraspecific genetic diversity provides further advantages for phylogeography. The simultaneous analysis of multiple genetic markers and study sites across a wide area is required in population genetics and phylogeography. Even if a single eDNA sample encodes the information of multiple individuals at a single sampling site, analyzing multiple eDNA samples from multiple study sites is required to provide the necessary information to reveal a species’ phylogeography. Furthermore, multiplex PCR amplification of different DNA regions using multiple primers can enable the simultaneous sequencing of multiple regions, making phylogeographic analysis even more accurate than PCR targeting a single region. Advances in eDNA technology are expected to promote inexpensive and non-invasive fish monitoring [144].

At present, various techniques, including conventional PCR, quantitative PCR, digital droplet PCR, and metabarcoding methods, are used to detect fish eDNA in rivers, lakes, ponds, reservoirs, and oceans [121, 127, 145–148]. Fish eDNA approaches are powerful tools for non-invasive monitoring of fish fauna in Water Framework Directive [149]. Moreover, they are quickly becoming recognized as essential tools for aquatic ecosystem restoration planning through the regular monitoring of species composition, with a focus on the detection of indicator organisms [150]. For example, eDNA analysis has been performed to investigate the presence of Mediterranean fish in the Arab Gulf. This species is considered invasive and could harm the native elasmobranchs that settled there hereafter [151]. Moreover, the paleolimnological history of threatened freshwater fish can be reconstructed using species diagnostic markers amplified from eDNA, allowing determination of the colonization history of freshwater fish and the structure of ecosystems, thereby aiding in the identification of native diversity and the introduction of non-native species [152]. Owing to its high detection rate at low densities, eDNA-based monitoring is helpful for early detection and monitoring of invasive species and can further be used to inform fish management and enhance the likelihood of successful containment actions for invasive species [153].

## A Collection of Study Findings from Diverse Sources

In this section, we present our findings on fish diversity in Saudi Arabia using multiple methodologies (Table-1) [75, 154–166] and summarize the approaches to fish identification mentioned in the previous section (Table-2) [33, 103, 167–171]. The Red Sea in Saudi Arabia has a huge potential for biodiversity. In the past 5 years, many new fish have been reported to the international world. In 2020 and 2021, the reported fish still use the conventional morphological identification method [154, 155]. The conventional morphological method is the simplest and most basic identification method [33]. However, this method is still used due to the fast results, no chemicals required, and sometimes, the reported specimens are found unexpectedly and must be identified immediately. For example, the new specimens of *Schindleria* were collected during ichthyoplankton sampling in Red Sea, Jeddah, in 2019 [154]. The discovery of the fish was unexpected since it was found during another research (ichthyoplankton). If not immediately identified, the quality of the specimen will be decreased and difficult to observe. This method is also still used recently in identifying alien fish that have never been reported or found accidentally such as in Indonesia or Mediterranean water (Lebanon, Turkey, Greece, Algeria, Tunisia, Spain, and Slovenia) [172, 173]. However, this method has lower accuracy compared to the current method, so further research always combines morphological identification with molecular. In addition to conventional morphological methods, fish identification studies in Saudi Arabia have reported the use of NGS and DNA barcoding [156, 157]. This method is also widely applied in various Southeast Asian countries, Japan, Europe, and South America [174].

**Table-1:** Classification of fish diversity in Saudi Arabia using multiple methodologies.

Samples	Marker types	Outcomes	Locations	References
Conventional morphological identification				
*Schindleria parva*	n/a	It is distinguished by a lack of pigmentation on the body, an inconspicuous gas bladder, and short teeth on the premaxillae. The holotype is a female of 11-mm standard length (11.9 mm total length), while the paratype is a male of 9-mm SL. Dorsal fin rays 10 (9), anal fin rays 9 (7). The body depth at the pectoral-fin origin is 5% (4%) of SL, the depth at the anal-fin origin is 8% (7%) of SL, the predorsal length is 63% (65%) of SL, the preanal length is 72% (72%), and the first anal-fin ray is located below the fourth dorsal-fin ray, for a total of 23+16 myomeres.	Red Sea, Jeddah, Saudi Arabia	[154]
*Ophichthus olivaceus*		It is distinguished from its congeners by the following combination of characters: Vertebrae 141–145; tail moderately short (2.15 in TL); head short (9.6–11.1 in TL); uniserial teeth in jaws and on vomer; pectoral fins slightly elongate, not lanceolate, with upper rays longer than lower; dorsal fin origin above middle of pectoral fin; and a generally uniform, dark tan body with an olivaceous hue shading to tan or pale orange ventrally, with two pale yellow blotches above the pectoral-fin base, snout and lower jaw dark brown, and olivaceous median fins.	Jizan, Red Sea coast of southern Saudi Arabia	[155]
*Priolepis melanops*		*Priolepis melanops* stands out from its congeners with the following characteristics: The species has unbranched dorsal-fin rays VI+I, 9, no elongated spines in the first dorsal fin, unbranched anal-fin rays I, 8, and pectoral-fin rays 14–15. The longitudinal scale series is 25, and there are no scales on the head or predorsal midline, but the sides of the nape are scaled. The sensory papillae below the eye have a developed transverse pattern. The fifth pelvic-fin ray is unbranched and 47% longer than the fourth ray. The body and most of the head are reddish-orange and coated with melanophores. The snout, mouth, chin, and chest are black, and the iris is black. The fins are translucent, with a faint black stripe down the base of each dorsal fin.	Al Lith, the coast of the Red Sea, Saudi Arabia	[159]
Next-generation sequencing				
*Oreochromis spilurus saudii*	Antimicrobial peptides (AMPs)	The genome assembly of the newly cultured marine subspecies *O. spilurus* (0.76 Gb) has been completed for the first time. These predicted peptides are involved in major cellular processes and aid in diagnosing various fish ailments. Furthermore, 262 potential AMPs were discovered, which could aid in practical molecular breeding and combating emerging bacterial and viral illnesses. This subspecies can easily survive in the Red Sea in Saudi Arabia (salinity 42 ppt), indicating that this tilapia has a high salt tolerance gene.	Seawater ponds at the Jeddah Fisheries Center on the Red Sea, Saudi Arabia	[75]
*Chaetodon austriacus*	Genomic DNA	Using existing bony fish (superclass *Osteichthyes*) genomes as a reference, 28,926 high-quality protein-coding genes were predicted from 13967 assembled scaffolds. The quality and completeness of the *C. austriacus* draft genome indicate that it has the potential to serve as a resource for studies on the co-evolution of reef fish adaptations to the unique Red Sea environment, as well as a comparison of gene sequences between closely related congeneric butterflyfish species distributed more broadly across the tropical Indo-Pacific.	Near Ablo Island, Saudi Arabia	[160]
		The authors used double-digest restriction site-associated DNA sequencing (RAD-Seq) data to discover single nucleotide polymorphism (SNP) markers and their related functions with and without our reference genome to investigate if it improves the quality of RAD-Seq. Our analyses show a modest difference in the number of no annotated versus annotated SNPs across all species, highlighting the benefit of using genomic resources for closely related but not distantly related butterflyfish species based on the ability to assign putative gene function to SNPs and an enrichment of genes related to calcium transmembrane transport and binding among sister butterfly-fish taxa.	Red Sea and Arabian Sea	[161]
DNA barcoding				
*Garra tibanica*	Cytochrome *b* gene	The clusters, inside groups, were supported by high bootstrap values and revealed that *G. tibanica* and *Garra sahilia* are related lineages in the same clade with 98.69% identity, and it is also consistent with traditional morphologically-based inferences.	Wadi Kadrah, Medina province, Saudi Arabia	[162]
*Epinephelus tauvina*	Genomic DNA from caudal fin	These findings could considerably impact grouper conservation and genetic improvement efforts. Intersimple sequence repeat (ISSR) and microsatellite (SSR) markers were beneficial for studying grouper species’ genetic diversity and structure.	Khafji, Al-Jubail, Al-Qatif, and Salwa, Saudi Arabia	[163]
*Epinephelus coioides*				
*Epinephelus malabaricus*				
*Epinephelus bleekeri*				
*Epinephelus areolatus*				
Genera from *Cyprinion*	Cytochrome *b* gene	The first cytochrome b gene sequences of *Cyprinion acinaces* acinases and *Carasobarbus aponesis* were identified and deposited in the public Gene Data Bank. The phylogenetic tree separated the species into two primary clusters, each with four sub-clusters. The evolutionary study validates the early taxonomic categorization and confirms that *Cyprinion acinaes hijazi* is a subspecies of *Cyprinion acinaces acinaces*. The phylogenetic tree built from the cytochrome b sequence also showed that *Carasobarbus apoensis* found in Saudi Arabia, is genetically closely linked *to Carasobarbus luteus* .	Wadi Khadrah and Ain Al-Jamma in Khyber, Medina Region, Saudi Arabia	[164]
Genera from *Carasobarbus*				
*Chlorurus sordidus*	Genomic DNA	The current study found that C oxidase subunit I (*COI*) outperformed ISSR and start codon-targeted (SCoT) markers for discriminating across parrotfish species. Furthermore, ISSR outperformed SCoT since it could distinguish three unique groups in principal component analysis. This investigation also established the presence of three unique parrotfish species, providing insight into their diversity.	Farasan Islands on the Jeddah Coast, Saudi Arabia	
*Cheilinus trilobatus*				
*Cheilinus quinquecinctus*				
*Oreochromis niloticus*	Randomly amplified polymorphic DNA	i) *O. niloticus* harvested from H1, H2, and H3 showed the maximum genetic variation (99.99) caused by OPA-02, OPA-05, and OPA-08. ii) The maximum and minimum polymorphism was recorded as 99.99 and 63.40% by OPA-05 and OPA-09. iii) The authors recorded the highest genetic variation in *O. niloticus* collected from the H4 location and the lowest from H1, which indicates that fish from H4 have more heterozygous genotypes. iv) Genetic distance ranged between 0.0005 and 0.0996. The highest and lowest genetics were recorded in the fish stocks obtained from H1 and H2, respectively.	Wadi Hanefah, Riyadh, Saudi Arabia	[156]
*COI* sequences (DNA barcoding)				
*Siganus rivulatus*	Genomic DNA	The results for the SSR markers revealed seventy polymorphic alleles, with an average of 5.83 alleles per locus. Furthermore, the interpopulation genetic diversity was 0.063. The nucleotide content of the MT-*COI* sequences showed significant differences between the two examined populations. Phylogenetic analysis revealed that Red Sea samples were more flexible than Mediterranean Sea samples. The results suggested that the mtDNA of *S. rivulatus* is quite variable and a species-sensitive marker to detect probable genetic alterations, which could be part of the ecological adaptation and important to the success of the migrant *S. rivulatus* in the Mediterranean Sea.	Red Sea and the Mediterranean Sea	[157]
*Ophichthus olivaceus*	Mitochondrial *COI*	This finding highlights the benefit of combining all available sequence data for mitochondrial *COI* from *Ophichthinae*. This approach provides a comprehensive picture of evolutionary diversity at the species level. It allows for identifying close phylogenetic relationships, even when species IDs are uncertain due to barcoding data. The phylogenetic analysis identified *Ophichthus lithinus* as the closest relative of *Ophichthus olivaceus* spp. nov. among the 90 *Ophichthinae* species analyzed in this study. Bootstrapped studies revealed that the novel Red Sea species and *O. lithinus* formed reciprocally monophyletic clades.	Jizan, Red Sea coast of southern Saudi Arabia	[155]
Environmental DNA (eDNA)				
Fish from the Labridae family	DNA from seawater	The authors discovered a wide range of prominent, cryptobenthic, and commercially important reef fish at the genus level, with specific genera in the *Labridae* family over-represented. Our method, however, failed to capture a significant fraction of the fish fauna known to inhabit the Red Sea, which we ascribe to insufficient spatial sampling, amplification stochasticity, and an apparent lack of sequencing depth. Given the growth in fish species descriptions, the completeness of taxonomic checklists, and the improvement in species-level assignment using bespoke genetic databases demonstrated here, we believe the Red Sea region is excellent for further testing of the eDNA technique.	Arabian Sea and Oman Sea	[158]
Marine finfish	n/a	The authors explore using RNA-guided immunity to combat *Chillodonella* protozoan and nervous necrosis virus in marine finfish. In addition, they also emphasize the immunological application of CRISPR-Cas against bacterial illnesses in channel catfish	n/a	[166]
Channel catfish				

**Table-2:** Summary of approaches for fish identification.

Advantages	Disadvantages	References
Conventional morphological identification		
i) Low cost ii) Fast and easy technique iii) No chemical requires iv) No advanced laboratory equipment is required	i) Depending on the taxonomist’s experience ii) High subjectivity iii) Difficult to identify eggs and larvae iv) Requires whole fish sample	[33]
Next-generation sequencing		
i) High accuracy ii) Allowed the genome-wide and/or transcriptome-wide detection and characterization of genetic markers, microsatellites, and single nucleotide polymorphisms iii) Can identify eggs and larvae iv) No need for a whole fish sample v) Can identify larger mixed species v) Less time and cost than previous sequencing method	i) Chemical may be expensive ii) Complex iii) Need advanced laboratory equipment	[168, 169]
Nanopore sequencing		
i) High accuracy ii) Can identify eggs and larvae iii) No need for a whole fish sample iv) Can identify mixed species v) Less time and cost than the Sanger and Illumina sequencing method	i) Chemical may be expensive ii) Complex iii) Need advanced laboratory equipment	[170]
DNA barcoding		
i) High accuracy ii) Can identify eggs and larvae iii) No need for a whole fish sample	i) Chemical may be expensive ii) Complex iii) More time-consuming than the morphological method iv) Only for the targeted gene v) Difficult for mixed fish species vi) Need advanced laboratory equipment	[103, 169]
Environmental DNA		
i) Can detect the presence or recent presence of species without direct observation or capturing whole or parts of an organ (sperm or urine can be used) ii) Less costly and non-invasive monitoring of fish in an environment iii) High accuracy	i) Chemical may be expensive ii) Need advanced laboratory equipment iii) Relatively new, diverse, and under continuous development	[167, 171]

Uniquely, advanced recent methods such as clustered regularly interspaced short palindromic repeats (CRISPR) nucleic acid detection, eDNA, and DNA microarrays have not been reported in Saudi Arabia. The CRISPR system was initially discovered in bacteria and archaea as a prokaryotic adaptive immune system that specifically recognizes and destroys foreign nucleic acid [175]. CRISPR methods can be used to detect aquatic eDNA [176]. The Cas proteins form complexes with CRISPR RNAs that recognize a specific nucleic acid target sequence: Cas12a-containing complexes recognize DNA, whereas Cas13a complexes recognize RNA. It offers high specificity, sensitivity, and ease of operation, making it a suitable approach for early detection and identification of such species [175]. The CRISPR method has been widely reported in the USA and Japan and has been followed by other countries such as China, Brazil, India, and Southeast Asian Countries [167]. The study demonstrated that the CRISPR/Cas12a system accurately detects invasive aquatic species without requiring direct observation. Not only CRISPR, eDNA identification method has also not been reported in Saudi Arabia. Information related to eDNA has been added as a complement to the review. Table-1 shows the information regarding eDNA application in Oman. A study in Oman, a country closest to Saudi Arabia, used eDNA to identify marine fish, and it needs to be tried for future studies [158].

In addition to CRISPR and eDNA, DNA microarrays need to be applied to identify fish in Saudi Arabia. DNA microarrays are an invaluable tool for conducting fish research and conservation efforts as well as for assessing fish health and monitoring the spread of diseases. Since their introduction in 1995, DNA microarrays have generated considerable interest among biologists. With its capacity to concurrently exhibit the expression of numerous genes, this tool proves to be a potent instrument for genetic investigation [177]. DNA microarray comprises numerous immobilized DNA fragments arranged in a standardized pattern on nylon membranes, silicon chips, or tiny glass microscope slides [178]. It is commonly called DNA chips, DNA biochips [179], or Fish chips. A DNA microarray can compare a reporter probe with a known sequence to the DNA obtained from an unknown source in the target sample. Species-specific DNA sequences can be integrated into a DNA microarray, enabling its usage for identification purposes. This method is both cost-effective and very accurate for identifying species. This method has been used in European countries and has been applied in the Northeastern Atlantic, North Sea, Baltic, Mediterranean, and Black Sea [180]. The use of advanced methods (DNA Microarrays, CRISPR, and eDNA) needs to be conducted in Saudi Arabia since they can detect the presence or recent presence of species without direct observation or capturing whole or parts of an organ (sperm or urine can be used), less costly and non-invasive monitoring of fish in an environment, and high accuracy.

## Conclusions and Prospects

Overall, the results of this review suggest that fish identification methods need to be further developed in the future. Researchers continue to strive to achieve increased cost efficiency, convenience, objectivity, and more accurate results, which will help with taxonomy, ecology, diversity, and sustainability. Application of the latest methods to provide information regarding their advantages and disadvantages is further required.

Real-time identification methods with high accuracy are required to develop more advanced methods in the future. Indeed, real-time identification is needed to monitor the presence of fish in nature. In particular, Saudi Arabia is expected to be able to use advanced methods such as CRISPR, an innovative and potent tool for editing genomes, and DNA microarrays, which allow species-specific DNA sequences to be integrated into a DNA microarray, enabling its use for identification purposes. These techniques could provide more up-to-date biodiversity and ecological information that is useful for environmental sustainability.

## Authors’ Contributions

MBS: Conceptualization, literature review, and manuscript writing. MARN: Literature review and manuscript writing. NRD, MA, PWT, HIP: Literature review and manuscript writing. LAS, LS, MAAE: Supervision. All authors have read, evaluated, and approved the final version of the manuscript.
